# Immune Protective Evaluation Elicited by DNA Vaccination With *Neospora caninum* Dense Granules Proteins in Mice

**DOI:** 10.3389/fvets.2021.638067

**Published:** 2021-02-26

**Authors:** Guili Yu, Wei Liang, Qiankun Yang, Jinxin Wang, Yu Wang, Tianmeng Zhang, Xiao Zhang, Hui Fan, Panpan Zhao, Lili Cao, Jingquan Dong

**Affiliations:** ^1^Jiangsu Key Laboratory of Marine Biological Resources and Environment, Jiangsu Key Laboratory of Marine Pharmaceutical Compound Screening, Co-Innovation Center of Jiangsu Marine Bio-industry Technology, Jiangsu Ocean University, Lianyungang, China; ^2^Department of Laboratory Medicine, the Second People's Hospital of Lianyungang City, Lianyungang, China; ^3^Department of Parasite, Jilin Academy of Animal Husbandry and Veterinary Medicine, Changchun, China; ^4^Key Laboratory of Zoonosis, Ministry of Education, College of Veterinary Medicine, Jilin University, Changchun, China

**Keywords:** *Neospora caninum*, DNA vaccines, dense granules proteins, immune responses, protective efficacy

## Abstract

*Neospora caninum*, an obligate intracellular protozoan, is the major cause for neosporosis and brings serious economic losses to cattle breeding industries worldwide. After invasion, dense granules proteins are abundantly secreted and being important components of parasitophorous vacuole and intravacuolar network where *N. caninum* survives and replicates. The aim of the present study was to evaluate the protective immunity induced by DNA vaccines with genes encoding dense granules proteins 1 (GRA1), GRA4, GRA9, GRA14, GRA17, and GRA23 against *N. caninum* tachyzoites in BALB/C mice. Eukaryotic expressing plasmids of pcNcGRAs were constructed and the mice were intramuscularly immunized with pcNcGRAs followed by challenging infection with lethal doses of *N. caninum*. Immune responses were evaluated through monitoring the levels of serum antibodies, measurement of lymphocyte proliferation, and secretion of cytokines. Immune protection assays were carried out through monitoring survival time, body weight, and parasite burden in the brains. Results showed that all the pcNcGRA DNA vaccines could trigger remarkably specific humoral and cellular responses, with higher levels of IgG and IgG2a antibodies as well as obviously increased secretion of Th1-type IFN-*γ* cytokines. The immune protective efficacy revealed that pcNcGRA4, pcNcGRA14, and pcNcGRA17 DNA vaccines could individually increase the survival rate to 50, 37.5, and 25% in comparison with 0% in the control group; prolong the survival time more than 20.88 ± 11.12, 18.88 ± 10.83, and 16.63 ± 10.66 days compared with the control group of 4 ± 1.31 days; and decrease parasite burden in the brains to 297.63 ± 83.77, 471.5 ± 110.74, and 592.13 ± 102.2 parasites/100 ng comparing with 1221.36 ± 269.59 parasites/100 ng in the control group. These findings indicated that NcGRA4, NcGRA14, and NcGRA17 are potential vaccine candidates; NcGRA4 displayed better performance in immune protective efficacy and could be further combined with other advantageous antigens applied to the development of safe and effective DNA vaccines against *N. caninum*.

## Introduction

*Neospora caninum*, classified as an obligate intracellular protozoan parasite of phylum *Apicomplexa*, is the major pathogen for neosporosis in cattle and dog breeding industries (1–3). Susceptible animals become infected by ingestion of oocyst-contaminated foods or vertical transmission via placenta (4). *N. caninum*–infected dogs showed nervous system dysfunction and pregnant cows faced higher risks of reproductive failure, abortion, and neonatal mortality (5). According to statistics, *N. caninum* infection in cattle industries is causing dramatic negative economic losses ranging from US $43.08 billion to US $320.98 billion each year all over the world (6, 7). As one of the cattle-breeding countries, China is also facing challenges of continuous economic losses and paying much attention to neosporosis. Nowadays, sulfonamides are still widely used to ease neosporosis and control its spread. However, large negative effects and drug residues in animals are inevitable to threaten the breeding industries (5). Overall, development of safe and effective vaccines to disrupt invasion or persistent infection of *N. caninum* is urgently needed to ensure the healthy development of cattle industries.

Vaccines based on live, attenuated or killed parasites, and recombinant antigens are available for varieties of diseases through triggering immune responses. Both protective humoral and cellular immune responses involve in attenuating the pathogenicity of neosporosis. Substantial evidence shows that T helper (Th) 1-type immune response dominated by secretion of gamma interferon (IFN-γ), interleukin (IL)-12 cytokines, and production of IgG2a antibody is not only vital for acute *N. caninum* infections but is also an effective protection for chronic infections (8, 9). Also, Th2-type immune response triggered by antibody-mediated immunity is mainly functioned in combating *N. caninum* circulating in the blood or body fluids via activating complement system and limits the development of neosporosis (10, 11).

In *Toxoplasma gondii*, a closely related parasite to *N. caninum*, multiples DNA vaccines have already been developed and verified to be promising in the prevention of toxoplasmosis, such as dense granules proteins 1 (GRA1), GRA4, GRA7, and GRA14, for they could activate high levels of humoral and cellular protective immunity for relative long periods (12–14). For *N. caninum*, many studies have proposed potential antigens against neosporosis, such as surface antigens proteins 1 (SAG1) and SAG4 (15, 16); GRA1, GRA2, GRA6, and GRA7 (17–20); rhoptries proteins 2 (ROP2) and ROP40 (21–23); and micronemes proteins 1 (MIC1), MIC3, and MIC4 (24–26). However, research on DNA vaccines against neosporosis is still limited.

After invasion into host cells, *N. caninum* could secrete large amounts of GRA proteins, which are closely associated with its survival and intracellular replication (27). Many GRAs involved in the formation of parasitophorous vacuole and intravacuolar network structures, maintaining intracellular growing environment for tachyzoites. Moreover, GRAs are also found to exist in the cyst wall surrounding slowly dividing bradyzoites (7). Previous studies have verified that highly antigenic molecules of GRA2, GRA6, and GRA7 are effective vaccine candidates in *N. caninum* (20, 28, 29). In contrast, abundant GRA proteins secreted from *T. gondii* have been applied in immune protection and used as diagnostic indicators (30). Although many GRA proteins have been identified, few studies other than GRA2, GRA6, and GRA7 focus on studying its immune protection effects (31–35).

The present study aims to screen excellent DNA vaccine targets by evaluating humoral and cellular immune responses triggered by various reported *N. caninum* GRA proteins, including GRA1, GRA4, GRA9, GRA14, GRA17, and GRA23, through individual immunization with eukaryotic plasmids of pcNcGRA1, pcNcGRA4, pcNcGRA9, pcNcGRA14, pcNcGRA19, and pcNcGRA23 in BALB/c mice, and its immune protective efficacy after challenging with lethal doses of *N. caninum* tachyzoites. Our findings would lay a foundation for future research on providing new targets for DNA vaccine development.

## Materials and Methods

### Ethics Statement

All the animal experiments were approved by the Animal Welfare and Research Ethics Committee of Jilin University (permission number: pzpx20190929065). Six- to eight-week-old female BALB/C mice were maintained with SPF-level food and water *ad libitum* under environment conditions of light and dark cycle every 12 h in rearing isolators. The mice were monitored every 12 h and deaths were recorded. To minimize suffering, mice were humanely euthanized by cervical dislocation after anesthetization by subcutaneous injection of atropine (0.02 mg/kg) and then inhalation of CO_2_ (10–15 s) when they exhibited signs of illness and lost 20% body weight.

### Parasites

The Nc-1 strain of *N. caninum* tachyzoites were propagated by continuous passage in monolayer bovine kidney epithelial (MDBK; ATCC, USA) cells, which were cultured in Dulbecco's modified Eagle's medium (Biological Industries, Israel) and supplemented with 10% fetal bovine serum (Biological Industries), 100 U/ml penicillin, and 100 μg/ml streptomycin at 37°C in 5% CO_2_ conditions. To obtain enriched and purified tachyzoites for the following experiments, Nc-1 strain infected cells were collected at the stationary growth phase, released with a 27-gauge needle (Millipore, USA), purified with Percoll (Millipore), and enriched in the phosphate-buffered saline to a final concentration of 1 × 10^8^ parasites/ml.

### Preparation of *N. caninum* Tachyzoite Lysate Antigen

*N. caninum* tachyzoite lysate antigen (NLA) was prepared as follows: purified tachyzoite suspension was treated with Protease Inhibitor Cocktail (Sangon, Shanghai) and then lysed going through three cycles of freezing at −80°C and thawing at 4°C followed by ultrasonicaton on ice. Then, the mixture was centrifuged at 10,000 × *g* at 4°C for 30 min and supernatants were collected. After filtering through a 0.22-μm sterilized membrane (Merck Millipore, USA), NLA was stored at −80°C for further use.

### Construction of Recombinant pcNcGRA Plasmids and Expression *in vitro*

Total RNA was extracted from purified tachyzoites using RNAiso Plus (Monad, Wuhan), genomic DNA was removed, and cDNA was synthesized by the MonScript RTIII Super Mix with dsDNase (Two-Step) (Monad) according to the instructions. Specific amplification primers of NcGRAs were designed and listed in Table 1. Restriction enzyme cutting sites of *BamH*I and *EcoR*I were individually introduced into the 5′ ends of forward and reverse primers of NcGRA1. Restriction enzyme cutting sites of *EcoR*I and *Xhol*I were individually added to the 5′ ends of forward and reverse primers of NcGRA4, NcGRA14, NcGRA17, and NcGRA23. Restriction enzyme cutting sites of *Hind*III and *Bam*HI were individually added to the 5′ ends of forward and reverse primers of NcGRA9. Meanwhile, sequences of HA tag (TACCCATACGATGTTCCAGATTACGCT) were added into reverse primers after enzyme cutting sites. Target PCR products were obtained using PCR assays with Pfu DNA Polymerase (TIANGEN, Beijing) and then cloned into pMD18-T vector (Takara, Dalian) according to the manufacturer's manual. After sequencing, positive recombinant plasmids were cleaved with individual restriction enzymes (New England BioLabs, USA) and linked with linearized eukaryotic expression vector pcDNA 3.1(+) cleaved with same enzymes using T4 DNA ligase (Takara). After identification by restriction analysis, the positive recombinant expression plasmids of pcNcGRAs were purified using HiPure Plasmid EF Mega Kit (Magen, Guangzhou) and concentrations were measured on a Nanodrop ND-2000 apparatus (Thermo Scientific, USA). The eukaryotic recombinant plasmids of pcNcGRAs were diluted in sterile endotoxin-free PBS to a final concentration of 1 mg/ml and stored at −20°C for further use.

**Table 1:** Primer sequences used for amplification of NcGRAs genes.

**Name**		**Sequence (5′-3′)**	**Gene ID**
NcGRA1	F	ATAGGATCCATGGTGCGTGTGAGCGCT	NCLIV_036400
	R	GCGGAATTCTTAAGCGTAATCTGGAACATCGTATGGGTAATGTTGCCCTTGAAGAGC	
NcGRA4	F	GCGGAATTCATGGCAATGAAGGGTCTCTTCTTT	NCLIV_054830
	R	GTACTCGAGTTAAGCGTAATCTGGAACATCGTATGGGTAATGGCGCATTGCTTTCAA	
NcGRA9	F	GAGAAGCTTATGGCAATGAGGTCATTCAAGTCC	NCLIV_066630
	R	GCGGGATCCTTAAGCGTAATCTGGAACATCGTATGGGTATATTTCTCCGTTATGGTT	
NcGRA14	F	ATAGAATTCATGGCAATGCAGGGCGCAAC	NCLIV_016360
	R	ATACTCGAGTTAAGCGTAATCTGGAACATCGTATGGGTAGTAGACCGAGTTACCTGA	
NcGRA17	F	TATGAATTCATGGCAATGCGAGTGTGCGGTTCCT	NCLIV_005560
	R	ATACTCGAGTTAAGCGTAATCTGGAACATCGTATGGGTACTGGTTGCCACTGCCGGA	
NcGRA23	F	ATAGAATTCATGGCAATGCTCGCGTCCGCCGA	NCLIV_006780
	R	ATACTCGAGTTAAGCGTAATCTGGAACATCGTATGGGTAGTTCTTTCGCGCGAGCAC	

*F, forward; R, reverse. The underlined sequences represented restriction endonuclease recognition sites*.

Plasmid of pcNcGRAs were transiently transfected into RAW264.7 (ATCC, USA) cells previously prepared on coverslips in 24-well plates using Lipofectamine 2000 Transfection Reagent (Invitrogen, USA), and empty pcDNA 3.1(+) vector was used as negative control. After 72 h, cells were washed three times with PBS and fixed in 4% paraformaldehyde at room temperature (RT) for 10 min. After permeabilization in 0.1% Triton X-100 at RT for 20 min, cells were then washed with PBS three time times and blocked in 5% BSA at RT for 2 h. Next, cells were incubated with mouse anti-HA-Tag mAb (1:200; ABclonal, Wuhan) at 4°C overnight and fluorescein (FITC)-conjugated Affinipure Goat Anti-Mouse IgG(H + L) (1:100; Proteintech, Wuhan) at 37°C for 1 h, respectively. After washing unconjugated antibodies, cells were viewed on a fluorescence microscopy (Olympus, Japan).

### Mice Immunization and Infection

**Figure 1:**
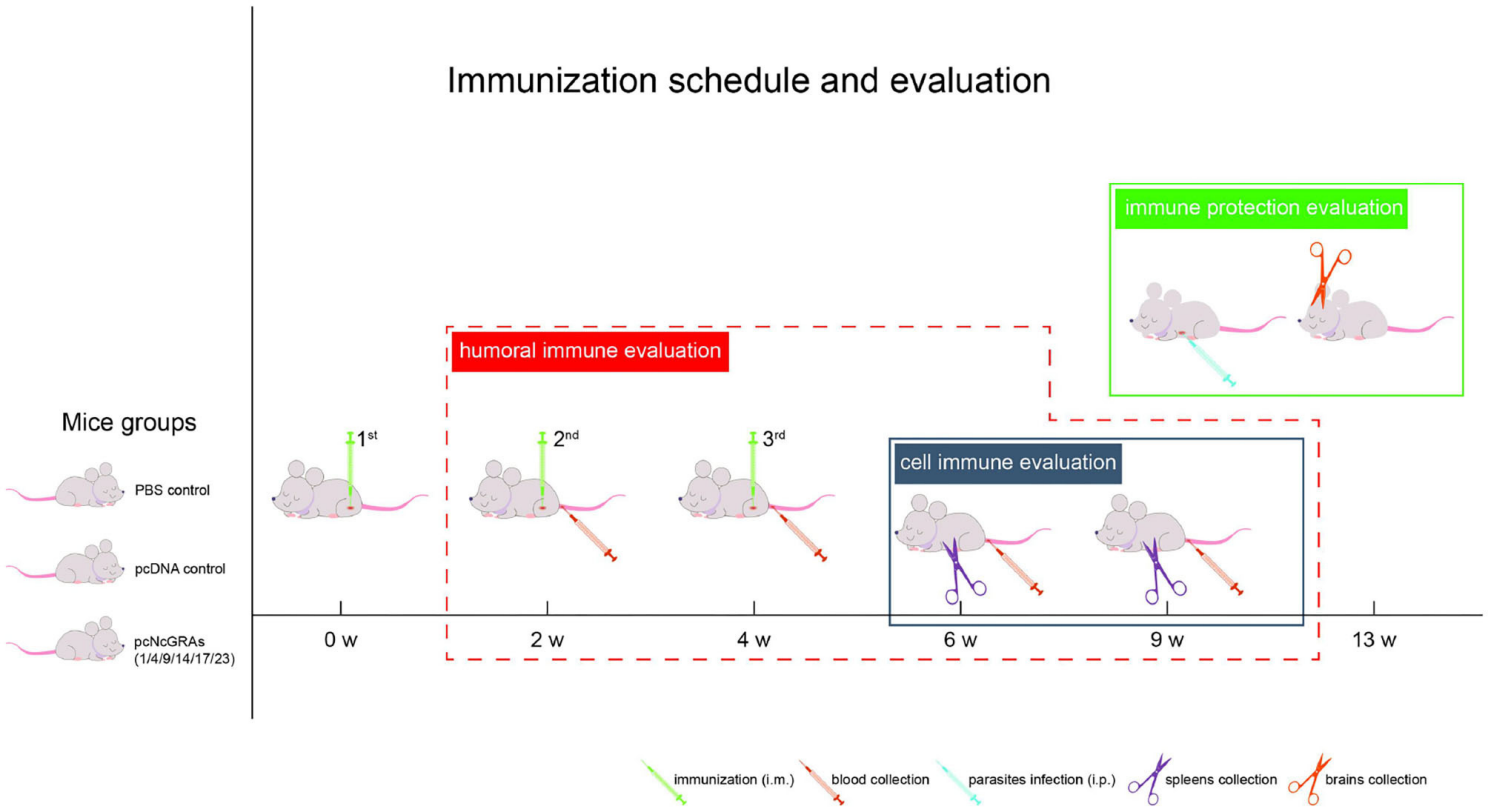
Immunization schedule and evaluation of protective efficacy. One hundred and twenty-eight BALB/c mice were randomly divided into 8 groups (16/group), including PBS control group, pcDNA control group, and pcNcGRA1, pcNcGRA4, pcNcGRA9, pcNcGRA14, pcNcGRA17, and pcNcGRA23 experimental groups. Mice were immunized three times with 2-week intervals. Four blood samples were collected at each time points of weeks 1, 4, 6, and 9 for humoral immune evaluation. Four spleen tissues were isolated at weeks 6 and 9 for cell immune evaluation. Eight BALB/c mice from each group inoculated with *N. caninum* tachyzoites at weeks 9 for immune protection evaluation.

As shown in Figure 1, BALB/c mice were randomly divided into 8 groups (16/group). Control groups were either injected with 100 μl of sterile PBS or 100 μg of pcDNA 3.1(+) plasmid. Experimental groups were vaccinated with 100 μg of pcNcGRA plasmids through intramuscular injection (anterior tibial muscle). This immunized protocol was conducted at weeks 0, 2, and 4. Whole blood was collected through tail-bleed at weeks 2, 4, 6, and 9, and sera were separated and used for humoral antibody assay. At weeks 6 and 9, four mice from each group were euthanized; spleens were removed and used for lymphocyte proliferation assay and cytokine measurement assay. The remaining eight mice were infected with 2 × 10^7^ *N. caninum* tachyzoites at weeks 9 through intraperitoneal injection while monitoring the survival time for 30 days, and brain tissues were isolated for parasite burden assays.

### Humoral Antibody Levels Assay

The specific antibody levels against NLA were monitored at weeks 2, 4, 6, and 9 in each group. NLA (100 μg/ml) was coated in 96-well plates (100 μl per well) at 4°C overnight. The plates were then blocked with 5% skim milk at RT for 2 h. Then, 100 μl of serum (100-fold dilution in PBS) was added into wells and incubated at 37°C for 2 h. Then, unconjugated antibodies were removed from wells through washing three times with PBST buffer and the remaining antibodies were detected with 100 μl of detection secondary antibodies of HRP-conjugated goat anti-mouse IgG, IgG1, or IgG2a (1:2,000; Abcam, UK) at 37°C for 1 h. After washing three times with PBST, 100 μl of TMB substrate solution (Beyotime, Beijing) was added into each well and incubated at 37°C for 15 min. The reaction were stopped by 100 μl of 2 M H_2_SO_4_ per well and OD_450nm_ values were measured on a microplate reader (BioTek, USA).

### Lymphocyte Proliferation Assay

Lymphocyte proliferation assay was carried out by isolation of spleens at weeks 6 and 9 (four mice per time-point and per group). Single-cell suspensions were prepared as previously described (20). Splenocytes were seeded into a 96-well plate at a density of 3 × 10^5^ cells per well and cultured in RPMI 1640 medium with 10% fetal bovine serum (Biological Industries). Cells were then stimulated with NLA at a final concentration of 10 μg/ml, concanavalin A (ConA, 5 μg/ml; Sigma, USA) as positive control, or non-treatment as negative control at 37°C/5% CO_2_ for 72 h. The supernatants were discarded and replaced with fresh medium containing 10 μl of Cell Counting Kit-8 solution (CCK8; Beyotime). After incubation at 37°C/5% CO_2_ for 3 h, the OD_450nm_ values were measured on a microplate reader (BioTek).

### Cytokine Measurement Assay

Cytokine levels of IL-4 and IFN-γ in the splenocyte culturing surpernatants were measured using IFN gamma or IL-4 Mouse ELISA Kit (Invitrogen, USA) according to the manufacturer's instructions. The OD_450nm_ values were read and converted to picograms per milliliter. Concentrations were calculated based on the standard curves generated in each plate.

### Determination of Parasite Burden

Parasite burden was measured in brain tissues from immunized and infection mice at weeks 9 (eight mice per group) using real-time quantitative qPCR method. Genomic DNA was extracted using TIANamp Genomic DNA Kit (TIANGEN, China) according to instructions and standard curve was established with serially diluted *N. caninum* tachyzoite Nc-1 strain DNA samples (1.0 × 10^2^-1.0 × 10^7^ parasites/ml). The parasite burden was measured by amplifying the Nc5 gene (36) and parasite burden in 100 ng of tissue samples was calculated.

### Statistical Analysis

Statistical analyses were conducted using SPSS (version 21.0; SPSS, Chicago, IL). All data represented mean ± SDs. A one-way ANOVA followed by Levene's test was used to analyze the difference between immunized groups and control groups or among different immunized groups. Correlation analysis was evaluated using non-parametric Spearman correlation with two-tailed tests. Kaplan–Meier survival curves were analyzed using log-rank (Mantel–Cox) tests. Differences were regarded as significant at ^*^p < 0.05, ^**^p < 0.01, or ^***^p < 0.001. Graphs were generated in GraphPad Prism 7.00 (GraphPad Software, La Jolla, CA, USA). All the experiments were performed three times with three technical replicates.

## Results

### Evaluation of Humoral Immune Response in Immunized Mice

**Figure 2:**
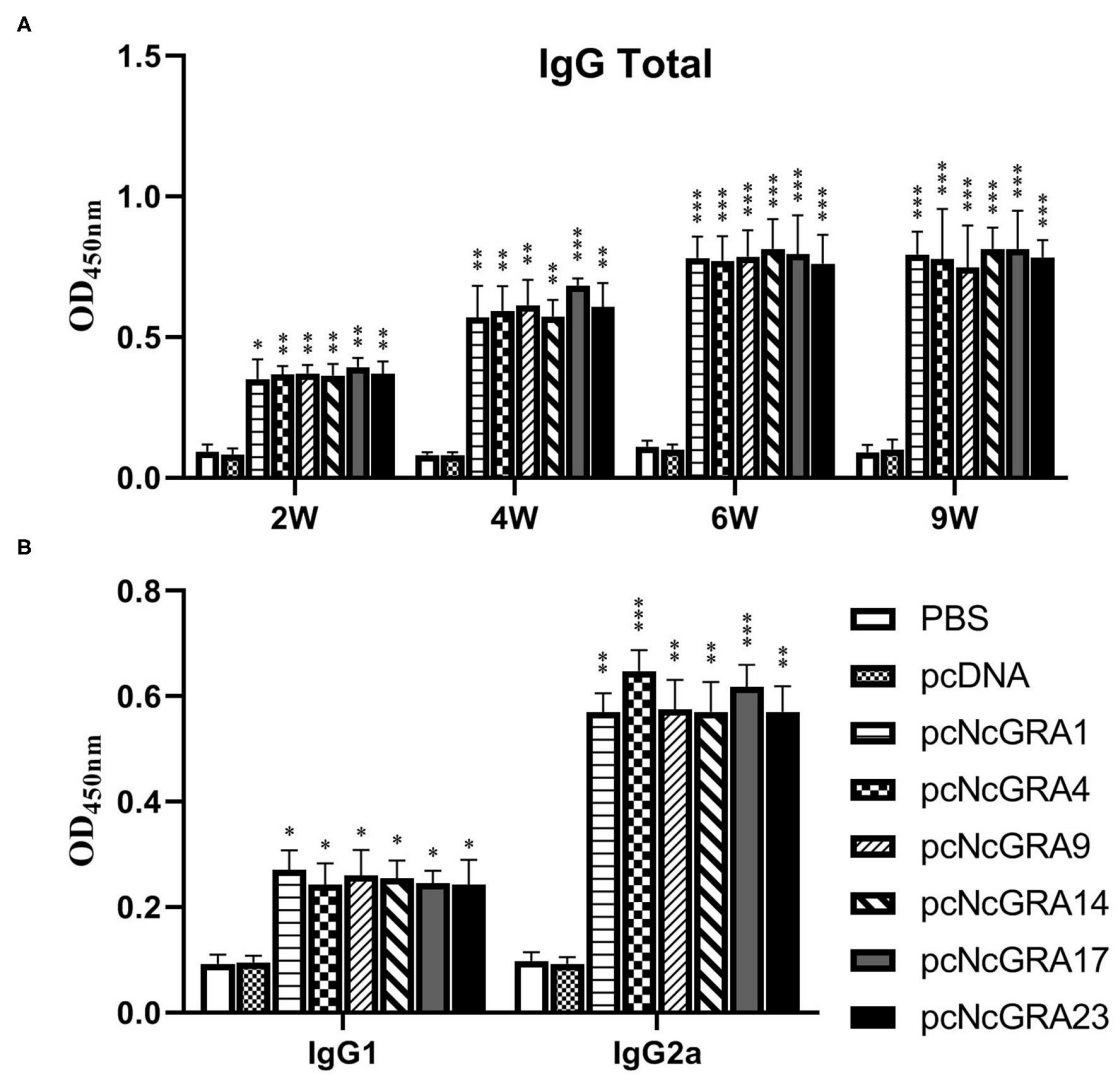
Time course assessment of humoral immune levels against pcNcGRAs in mice. **(A)** IgG total levels of serum from immunized and control mice at weeks 2, 4, 6, and 9. **(B)** IgG1 and IgG2a levels of serum from immunized and control mice at week 6. Each bar represented the mean ± SD from four mice per group. Significance was analyzed between experimental groups and the PBS control group and presented as ^*^p < 0.05, **p < 0.01, or ***p < 0.001.

After sequencing, positive recombinant plasmids of pcNcGRAs were individually transfected into RAW264.7 cells and proteins were successfully expressed *in vitro* (Supplementary Figure 1). To explore the humoral immune levels triggered by pcNcGRA DNA vaccines, serum was collected from mice tail at weeks 2, 4, 6, and 9. The IgG antibody levels against NLA were measured using indirect ELISA. As shown in Figure 2A, no significant differences were observed in the total IgG antibody levels when comparing serum from empty pcDNA 3.1(+) vector (pcDNA)-treated group and PBS-treated group throughout the monitoring periods from 2 to 9 weeks (*p* > 0.05), whereas each pcNcGRA-immunized group triggered remarkably increased levels comparing with the control group. The pcNcGRAs significantly triggered high IgG antibodies at week 2 (^*^p < 0.05 or ^**^p < 0.01), the levels were increased at week 4 (^**^p < 0.01 or ^***^p < 0.001), and the OD_450nm_ values peaked at week 6 (^***^p < 0.001). Moreover, the high antibody levels lasted for 5 weeks after the last immunization (^***^p < 0.001).

To identify the Type 1 T helper (Th1) and Type 2 T helper (Th2) cells stimulated by pcNcGRA DNA vaccines, the IgG1 and IgG2a antibody levels in serum were determined at week 6. As shown in Figure 2B, there was no obvious difference between the pcDNA-immunized groups and negative control group when measuring the OD_450nm_ values of IgG1 and IgG2a (*p* > 0.05). The IgG1 levels in pcNcGRA-immunized groups slightly increased (^*^p < 0.05), whereas the IgG2a levels remarkably upregulated (^**^p < 0.01 or ^***^p < 0.001) in comparison with that in the control groups. These data indicated that immunization with pcNcGRA DNA vaccines mainly induced Th1 immune responses in mice.

### Evaluation of Cell Immune Response in Immunized Mice

#### Lymphocyte Proliferation

**Figure 3:**
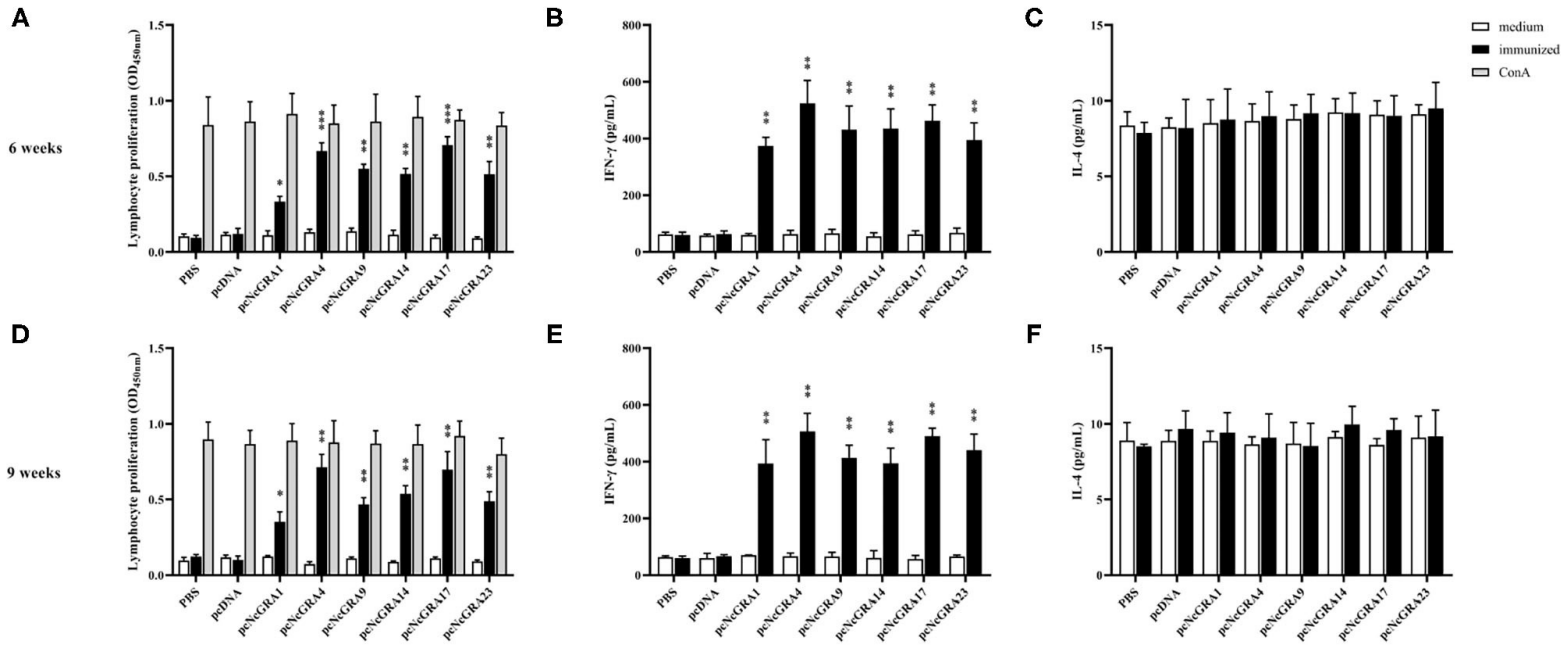
Assessment of cell immune levels by measuring splenocyte proliferative responses and cytokine production. **(A,D)** Splenocyte suspensions were prepared from immunized and control BALB/c mice (*n* = 4 per group) at week 6 or 9, and then stimulated with NLA, ConA as positive control, non-treatment was used as negative control. After 72 h, the cell proliferation was determined using CCK8 assays. **(B,E)** The supernatants were collected at 24 h for IL-4 measurement **(C,D)** and 96 h for IFN-γ measurement **(C,F)**. Each bar represents the mean ± SD from four mice per group. Significance was analyzed between experimental groups and the PBS control group and presented as ^*^p < 0.05, ^**^p < 0.01, or ^***^p < 0.001.

To assess the cell-mediated immune responses, spleens were isolated from mice after 2 and 5 weeks since the last immunization. Lymphocyte proliferation assays were conducted using ConA as positive control, NLA as stimulators, and medium as negative control. As shown in Figures 3A,D, mice immunized with PBS or pcDNA both triggered no statistically significant proliferative response (*p* > 0.05), by contrast, the pcNcGRA-immunized mice caused obviously high levels of lymphocyte proliferation both at week 6 and 9 after stimulation with 10 μg/ml of pcNcGRAs (^*^p < 0.05, ^**^p < 0.01, or ^***^p < 0.001) or 5 μg/ml of ConA (^***^p < 0.001). Overall, these results indicated that mice immunized with pcNcGRAs induced cell immune response.

#### Cytokine Production

**Figure 4:**
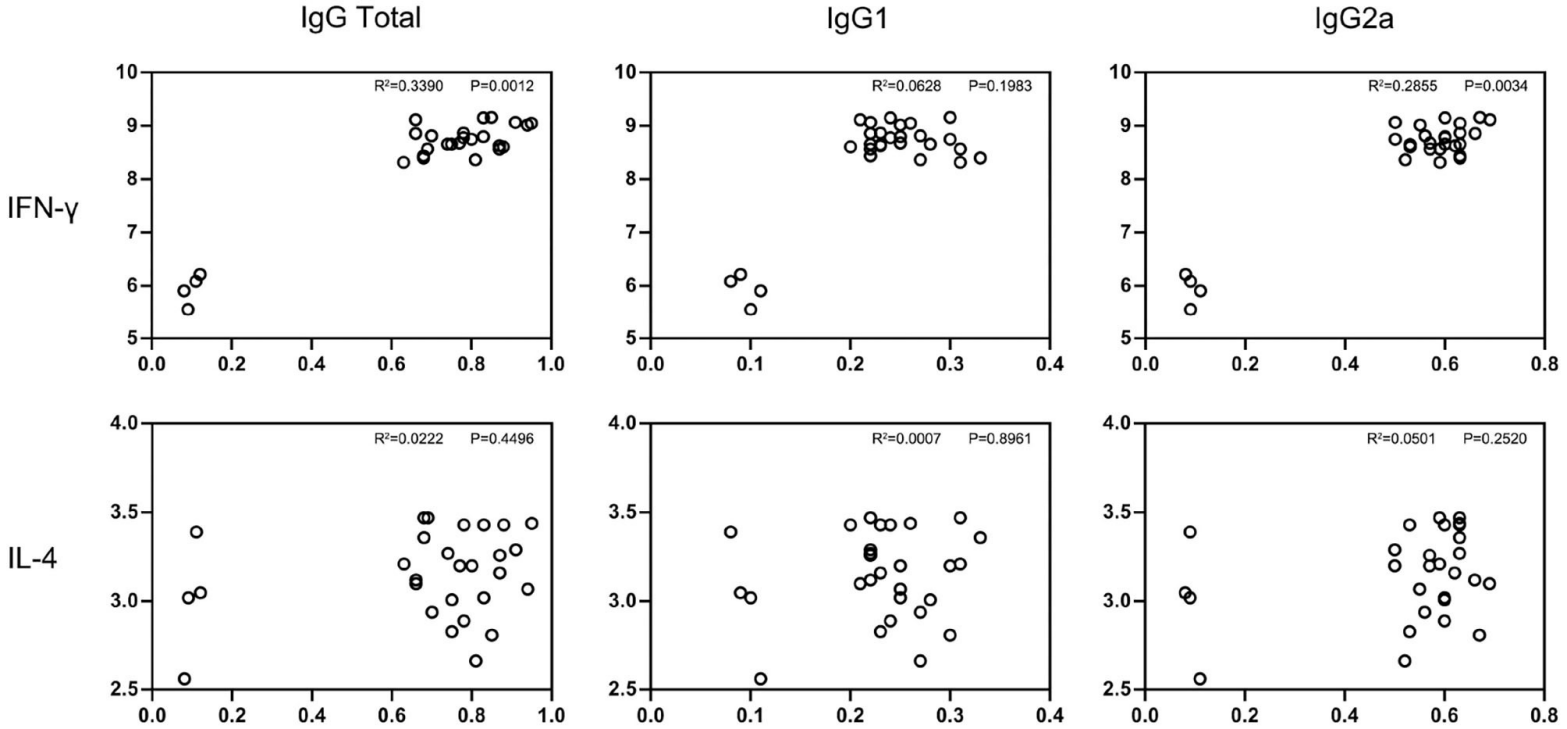
Correlation analysis between IgG antibody levels and cytokine production. Correlation between the antibody levels of IgG total, IgG1, IgG2a, and cytokine production of IFN-γ and IL-4 in immunized BALB/c mice were analyzed using non-parametric Spearman correlation with two-tailed tests. *P*-value represents the difference between the two groups and R square represents the correlation between two groups. *P* < 0.0332 indicates that the difference was significant.

The culturing supernatants were harvested from NLA-stimulated splenocytes for cytokine measurement. The secretion levels of Th1 (IFN-γ) and Th2 (IL-4) were determined and results are displayed in Figures 3B,C,E,F. All the splenocytes from pcNcGRA-immunized mice could induce high levels of IFN-γ (^**^p < 0.01), on the contrary, insignificant changes of IL-4 levels (*p* > 0.05). Furthermore, correlation analysis was performed to explore the relationship between humoral immune response and cellular immune response. As shown in Figure 4, significantly positive correlation not only occurred between IFN-γ secretion and IgG antibody levels (*p* = 0.0012, *r*^2^ = 0.3390) but also appeared between IFN-γ secretion and IgG2a antibody levels (*p* = 0.0034, *r*^2^ = 0.2855). There was a negative correlation between IL-4 and IgG1, but was not significant (*r* = −0.02586, *p* > 0.05). Overall, these data illustrated that all the pcNcGRAs triggered Th1 response.

#### Protective Efficacy Evaluation in pcNcGRA-Immunized Mice

**Figure 5:**
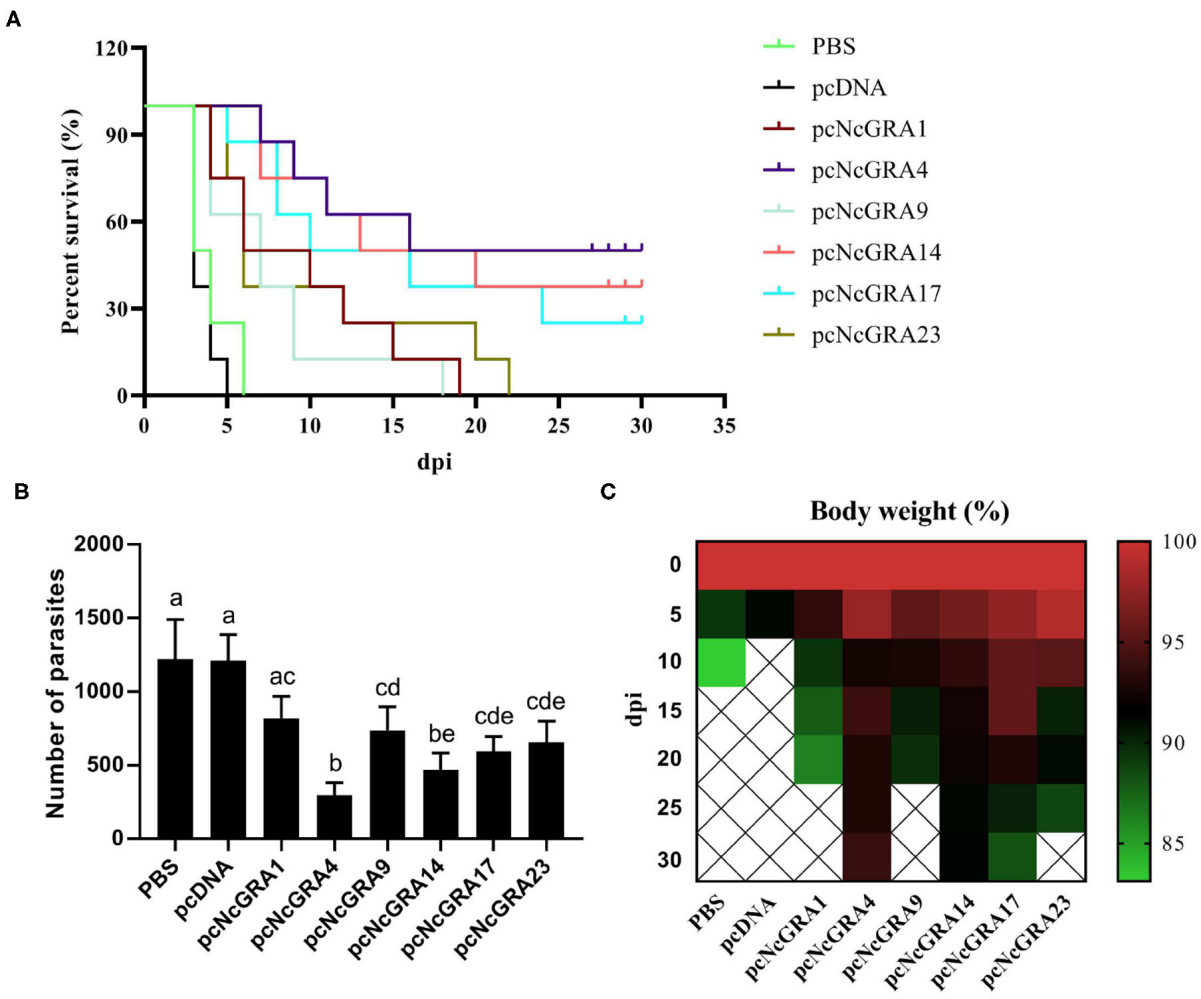
Evaluation of immune protection. After 5 weeks since the final immunization, eight BALB/c mice per group were challenged with 2 × 10^7^ *N. caninum* tachyzoites and the survival time was monitored **(A)**. Kaplan–Meier survival curves were analyzed using log-rank (Mantel–Cox) tests. **(B)** A total of 100 ng of brain tissues was used for measuring the parasite burden using qPCR method. **(C)** Body weight was monitored for 30 days after challenging. Differences were regarded as significant when indicated with different lowercase letters in different groups.

To assess the protective function of pcNcGRA vaccines, eight mice per group were infected with lethal doses of 2 × 10^7^ *N. caninum* tachyzoites and monitored the survival time for 30 days. As shown in Figure 5A, mice in control groups immunized with PBS or pcDNA died off within 5–6 days after infection. All the other immunized mice (pcNcGRA1: 9.5 ± 5.5 days, *p* = 0.0074; pcNcGRA4: 20.88 ± 11.12 days, *p* = 0.0001; pcNcGRA9: 7.75 ± 4.65 days, *p* = 0.0056; pcNcGRA14: 18.88 ± 10.83 days, *p* < 0.0001; pcNcGRA17: 16.63 ± 10.66 days, *p* = 0.0003; pcNcGRA23: 10.25 ± 7.03 days, *p* = 0.0074) displayed significantly longer survival time than the PBS-treated control groups (4 ± 1.31 days). During the monitoring periods, pcNcGRA4 and pcNcGRA14-immunized mice prolonged 10 more days of life and increased survival rate to 50 and 37.5%, respectively. In contrast, the death rates in pcNcGRA1 and pcNcGRA9-immunized groups were both 100%. For the pcNcGRA17 group, the death rate decreased to 75%. In addition, further analysis was made between different immunized groups. The pcNcGRA4-immunized mice showed significantly higher survival percentage than that in the pcNcGRA1 or pcNcGRA23 (^*^p < 0.05) and pcNcGRA9 (^**^p < 0.01). Moreover, pcNcGRA4-immunized mice showed significantly increased survival percentage than pcNcGRA1, pcNcGRA9, or pcNcGRA23 (^*^p < 0.05). Comparisons between other immunized groups manifested no obvious significance (*p* > 0.05).

The parasite burden in infected mice brain tissues was determined in Figure 5B. Comparing with the PBS-treated group (1221 ± 269.59 parasites/100 ng), numbers of parasites in pcNcGRA-immunized mice brain tissues were significantly reduced (pcNcGRA4: 297.63 ± 83.77 parasites/100 ng, *p* < 0.001; pcNcGRA9: 738.36 ± 159.42 parasites/100 ng, *p* = 0.029; pcNcGRA14: 471.5 ± 110.74 parasites/100 ng, *p* = 0.001; pcNcGRA17: 592.13 ± 102.2 parasites/100 ng, *p* = 0.005; pcNcGRA23: 656 ± 144.3 parasites/100 ng, *p* = 0.009) except for pcNcGRA1-immunized group (818.38 ± 150.18 parasites/100 ng, *p* = 0.096). Comparisons between immunized groups with low parasite burden presented that pcNcGRA9, pcNcGRA17, or pcNcGRA23 shared similar levels with pcNcGRA1 group (*p* > 0.05). It is worth mentioning that the pcNcGRA4-immunized mice shared the lowest levels of parasite burden, which were remarkably decreased than other immunized groups (^**^p < 0.01 or ^***^p < 0.001) except for pcNcGRA14 (*p* = 0.096). Meanwhile, body weight was also recorded throughout the whole survival period. As shown in Figure 5C, minimal body weight decrease in the survived mice occurred in the pcNcGRA4- or rNcGRA14-immunized groups compared with other groups. After comprehensive evaluation, these results illustrated that pcNcGRA4, pcNcGRA17, and pcNcGRA23 DNA vaccines could be used for protection against *N. caninum* infection, and pcNcGRA4 DNA vaccine elicited the best protection effects on infected mice and could improve the survival rate to 50% as well as decrease the parasite burden in brain tissues to 24.38%.

## Discussion

Neosporosis have threatened the cattle industries for a long time; however, no effective drugs or vaccines are available to use (5, 37). Up to now, there was only one licensed *Neospora* vaccine of Bovilis Neoguard composed of tachyzoite lysate available in partial countries (38). However, it has been stopped to be further used now for its uncompleted efficacy and risks of early embryonic death (39, 40). Along with the increasing demands for control and treatment with neosporosis, especially for countries with large numbers of cattle breeding industries, safe, and effective vaccines against neosporosis are urgently needed.

There have been several studies focused on live vaccines and subunit vaccines. In contrast, live vaccines shared excellent advantages of efficacy via triggering strong humoral and cellular immune responses; however, they were always regarded as alternatives and restricted its applications in the pharmaceutical industry for its high production costs, limitation in transportation and storage, and high risks for virulence return (41). Thus, vaccines of recombinant antigens are better choices for effective protection. In-depth knowledge of the underlying pathogenic mechanisms of *N. caninum* would help to combat with neosporosis. For apicomplexan parasites, GRA proteins are advantage weapons during invasion and construct the core contents of parasitophorous vacuole and intravacuolar network structure where parasites survive and replicate within host cells (32, 42). For instance, deletion of TgGRA6 gene would replace intravacuolar network with small vesicles and limit the replication of *T. gondii* (43). TgGRA2 gene disruption would inhibit Rab11A vesicle formation, disrupt intravacuolar network structure, and weaken the pathogenicity of *T. gondii* (44, 45). Moreover, many studies also proved to have protective effects in mice immunized with recombinant NcGRA antigens (20, 46).

Recently, DNA vaccines prepared with eukaryotic expression vectors could protect hosts from intracellular parasite invasion. Meanwhile, it shared advantages of low costs, easy transportation, and excellent immune protection through inducing lasted humoral and cellular responses (47). Many DNA vaccines have been evaluated in *T. gondii* by constructing with different antigens and verified to have the ability to trigger humoral and cellular immune responses lasting for long periods (48–50). For instance, TgGAR14 shares functions of intervacuolar transportation and mice immunized with TgGAR14 DNA vaccines would trigger specific immune responses, prolong the survival time, and reduce the parasite burden in target tissues (14). Abundant GRA proteins are secreted to make *N. caninum* survival and replication during invasion into hosts. Although many NcGRAs have been proved to be dominant antigens, there are few studies that focus on the evaluation of immune protection other than NcGRA1, NcGRA2, NcGRA6, and NcGRA7 up to now (17, 20, 28, 29). Ellis et al. (17) expressed GRA1 recombinant protein and evaluated the immunization effects using mice model. Results showed that GRA1 protein vaccine could produce protection in vertical propagation in mouse models (17). Considering that GRA1 protein was secreted after *N. caninum* invasion into host cells, the GRA1 DNA vaccine would stimulate a stronger Th1 immune response than GRA1 protein vaccine. Thus, GRA1 DNA vaccine was used as a positive control in our research. The present study evaluated the protective immunity of DNA vaccines encoding GRA1, GRA4, GRA9, GRA14, GRA17, and GRA23 of *N. caninum* in mice for the first time. Results demonstrated that mice immunized with these NcGRA DNA vaccines could both lead to humoral responses and cellular responses. Moreover, these vaccines exhibited different degrees of protective efficacy in survival time, body weight, and parasites in the brain tissues.

Humoral response plays vital roles in killing intracellular parasites (51). In the present study, the IgG total levels in serum isolated from pcNcGRA DNA–immunized mice were significantly upregulated than that in the PBS control group. In addition, the IgG secretion amount increases as time goes on and peaks at 2 weeks since the last immunization, and the antibody levels lasted for 5 weeks. Also, the IgG1 and IgG2a antibody levels were also measured after 2 weeks since the last immunization, and results demonstrated that pcNcGRA DNA–immunized mice could both upregulate the IgG1 levels (^*^p < 0.05) and dramatically increase the IgG2a antibody levels (^**^p < 0.01 or ^***^p < 0.001) in comparison with the control group. These data were in accordance with other DNA immunizations that trigger mixed Th1/Th2 immune response, and Th1 immune response occupies the predominant function with a higher level of IgG2a (51, 52).

The Th1-type immune response was closely related with the secretion of IFN-γ cytokine (9, 53). To further explore the relationship between IgG2a response and IFN-γ cytokine, splenocytes were individually isolated from pcNcGRA DNA–immunized mice and control mice and then stimulated with NLA *in vitro*. An obviously increased level of IFN-γ cytokine was generated followed by different pcNcGRA DNA immunization (^**^p < 0.01). Correlation analysis was also made and a significant difference occurred when comparing IgG total with IFN-γ or IgG2a with IFN-γ. The high levels of IFN-γ would contribute to enhance the macrophage function, disrupt parasitophorous vacuole through GTPases and p65 guanylate-binding proteins, and further eliminate the intracellular parasites (54, 55). Meanwhile, the Th2-type cytokine of IL-4 levels was also measured in stimulated splenocytes, however, there was no significance between all the pcNcGRA DNA–immunized groups and control group (*p* > 0.05). As expected, the *p-*values calculated from correlation analysis showed that no significant difference was observed between IgG and IL-4. These data indicated that immunization with pcNcGRA DNA vaccines could not cause sufficient B-cell proliferation and mast cell responses. This may be the main reason for the incomplete protection of mice from challenging with lethal dose of *N. caninum*.

T cell–mediated immunity mainly controls intracellular parasite infection. Splenocyte proliferation assays were carried out to determine the difference between immunized groups and the control group. A slightly increased level occurred in the pcNcGRA1 group (^*^p < 0.05); obviously upregulated levels existed in the pcNcGRA9, pcNcGRA14, and pcNcGRA23 groups (^**^p < 0.01); and an extremely significant increase occurred in the pcNcGRA4 and pcNcGRA17 groups (^***^p < 0.001).

The aforementioned results could only illustrate those pcNcGRAs that trigger humoral and cellular responses and each protective immune response still needed to be determined *in vivo*. In the present study, the immunized mice were further challenged with lethal doses of *N. caninum* after 5 weeks since the last immunization. Results showed that the longest survival time occurred in the pcNcGRA4-, pcNcGRA14-, and pcNcGRA17-immunized groups and the highest survival rate occurred in the pcNcGRA4-immunized group, which were consistent with the measurement of parasite burden in brain tissues. Surprisingly, pcNcGRA1-, pcNcGRA9-, and pcNcGRA23-immunized mice triggered high levels of humoral and cellular responses; however, their performance relatively weakened protective immunity with 100% death rates even though the survival time was prolonged to some extent. These data indicated that pcNcGRA4, pcNcGRA14, and pcNcGRA17 DNA have the priority to be chosen as candidate vaccines and pcNcGRA4 DNA vaccine performed the best. All these data were evaluated on the non-pregnant mice. Although evaluation of vaccines using this immunocompetent mice model is regarded as the gold standard to explore protective antigens against *N. caninum*, some effective antigens display poor immune protection abilities in vertical transmission (5). Also, vaccines that triggered a strong immune response in cattle may not be available to protect against abortion (56). Thus, the protective efficacy of these DNA vaccines on pregnant mice to simulate vertical transmission and susceptible animals of cattle or other livestock still need to be determined in the future. The protective efficacy may differ in different animal models, physiological status, adjuvant types, immune methods, and antigen candidates from different strains; thus, promotion of safe, and effective vaccines should take comprehensive factors into consideration.

## Conclusion

The present study evaluated the immunogenicity and potency of NcGRA1, NcGRA4, NcGRA9, NcGRA14, NcGRA17, and NcGRA23 using BALB/c mice as models. These eukaryotic expression vector constructed pcNcGRA DNA vaccines could trigger a strong Th1-type immune response accompanying with significantly increased levels of IgG2a antibodies and IFN-γ cytokine production *in vivo*. The immune protective efficacy revealed that pcNcGRA4, pcNcGRA14, and pcNcGRA17 DNA vaccines had relatively strong effects on prolonging the survival time and decreasing the parasite burden in brains. Although none of them completely resist the challenge of *N. caninum* infection, they would probably be useful for further vaccine development against *N. caninum* through combining multiple protective antigens.

## Data Availability Statement

The original contributions presented in the study are included in the article/Supplementary Material, further inquiries can be directed to the corresponding author/s.

## Ethics Statement

All animal experiments have been reviewed and approved by research ethics from the Animal Welfare and Research Ethics Committee of Jilin University (Permit No. pzpx20190929065).

## Author Contributions

JD and WL designed the research. GY, WL, QY, and JW conducted the research. GY, YW, TZ, XZ, and HF analyzed the data. PZ, QY, and WL wrote the article. JD and LC directed the project. All authors have read and approved the article.

## Conflict of Interest

The authors declare that the research was conducted in the absence of any commercial or financial relationships that could be construed as a potential conflict of interest.

## References

[1] Bjerkas I Mohn SF Presthus J. Unidentified cyst-forming sporozoon causing encephalomyelitis and myositis in dogs. Z Parasitenkd. (1984) 70:271–4. 10.1007/BF00942230 6426185

[2] Dubey JP Lindsay DS. A review of *Neospora caninum* and neosporosis. Vet Parasitol. (1996) 67:1–59. 10.1016/S0304-4017(96)01035-7 9011014

[3] Lindsay DS Dubey JP. Canine neosporosis. J Vet Parasitol. (2000) 14:57–70. 10.2147/VMRR.S76969

[4] Frenkel JK Smith DD. Determination of the genera of cyst-forming coccidia. Parasitol Res. (2003) 91:384–9. 10.1007/s00436-003-0969-4 14505041

[5] Marugan-Hernandez V. *Neospora caninum* and bovine neosporosis: current vaccine research. J Comp Path. (2017) 157:193–200. 10.1016/j.jcpa.2017.08.001 28942304

[6] Dubey JP Schares G Ortega-Mora LM. Epidemiology and control of neosporosis and *Neospora caninum*. Clin Microbiol Rev. (2007) 20:323–67. 10.1128/CMR.00031-06 17428888 PMC1865591

[7] Reichel MP Ayanegui-Alcérreca MA Gondim LP Ellis JT. What is the global economic impact of *Neospora caninum* in cattle - the billion dollar question. Int J Parasitol. (2013) 43:133–42. 10.1016/j.ijpara.2012.10.022 23246675

[8] Nishikawa Y. Towards a preventive strategy for neosporosis: challenges and future perspectives for vaccine development against infection with *Neospora caninum*. J Vet Med Sci. (2017) 79:1374–80. 10.1292/jvms.17-0285 28690279 PMC5573824

[9] Baszler TV Long MT McElwain TF Mathison BA. Interferon- and interleukin-12 mediate protection to acute *Neospora caninum* infection in BALB/c mice. Int J Parasitol. (1999) 29:1635–46. 10.1016/s0020-7519(99)00141-1 10608450

[10] Nishikawa Y Xuan X Nagasawa H Igarashi I Fujisaki K Otsuka H. Monoclonal antibody inhibition of *Neospora caninum* tachyzoite invasion into host cells. Int J Parasitol. (2000) 30:51–8. 10.1016/s0020-7519(99)00162-9 10675744

[11] Epperon S Brönnimann K Hemphill A Gottstein B. Susceptibility of B-cell deficient C57BL/6 (microMT) mice to *Neospora caninum* infection. Parasite Immunol. (1999) 21:225–36. 10.1046/j.1365-3024.1999.00223.x 10320620

[12] Ching XT Fong MY Lau YL. Evaluation of immunoprotection conferred by the subunit vaccines of GRA2 and GRA5 against acute toxoplasmosis in BALB/c mice. Front Microbiol. (2016) 7:609. 10.3389/fmicb.2016.00609 27199938 PMC4847622

[13] Jongert E Craeye SD Dewit J Huygen K. GRA7 provides protective immunity in cocktail DNA vaccines against *Toxoplasma gondii*. Parasite Immunol. (2007) 29:445–53. 10.1111/j.1365-3024.2007.00961.x 17727568

[14] Ahmadpour E Sarvi S Soteh MBH Sharif M Rahimi MT Valadan R. Enhancing immune responses to a DNA vaccine encoding *Toxoplasma gondii* GRA14 by calcium phosphate nanoparticles as an adjuvant. Immunol Lett. (2017) 185:40–7. 10.1016/j.imlet.2017.03.006 28286231

[15] Cannas A Naguleswaran A Müller N Eperon S Gottstein B Hemphill A. Vaccination of mice against experimental *Neospora caninum* infection using NcSAG1- and NcSRS2-based recombinant antigens and DNA vaccines. Parasitology. (2003) 126:303–12. 10.1017/s0031182002002895 12741509

[16] Marugán-Hernández V Ortega-Mora LM Aguado-Martínez A Jiménez-Ruíz E Alvarez-García G. Transgenic *Neospora caninum* strains constitutively expressing the bradyzoite NcSAG4 protein proved to be safe and conferred significant levels of protection against vertical transmission when used as live vaccines in mice. Vaccine. (2011) 29:7867–74. 10.1016/j.vaccine.2011.07.091 21816191

[17] Ellis J Miller C Quinn H Ryce C Reichel MP. Evaluation of recombinant proteins of *Neospora caninum* as vaccine candidates (in a mouse model). Vaccine. (2008) 26:5989–96. 10.1016/j.vaccine.2008.08.043 18789996

[18] Nishimura M Kohara J Kuroda Y Hiasa J Tanaka S Muroi Y. Oligomannose-coated liposome-entrapped dense granule protein 7 induces protective immune response to *Neospora caninum* in cattle. Vaccine. (2013) 31:3528–35. 10.1016/j.vaccine.2013.05.083 23742998

[19] Ramamoorthy S Sanakkayala N Vemulapalli R Duncan RB Lindsay DS Schurig GS. Prevention of lethal experimental infection of C57BL/6 mice by vaccination with Brucella abortus strain RB51 expressing *Neospora caninum* antigens. Int J Parasitol. (2007) 37:1521–9. 10.1016/j.ijpara.2007.04.020 17568587

[20] Fereig RM Shimoda N Abdelbaky HH Kuroda Y Nishikawa Y. Neospora GRA6 possesses immune-stimulating activity and confers efficient protection against *Neospora caninum* infection in mice. Vet Parasitol. (2019) 267:61–8. 10.1016/j.vetpar.2019.02.003 30878088

[21] Pastor-Fernández I Arranz-Solís D Regidor-Cerrillo J Álvarez-García G Hemphill A García-Culebras A. A vaccine formulation combining rhoptry proteins NcROP40 and NcROP2 improves pup survival in a pregnant mouse model of neosporosis. Vet Parasitol. (2015) 207:203–15. 10.1016/j.vetpar.2014.12.009 25579396

[22] Alaeddine F Hemphill A Debache K Guionaud C. Molecular cloning and characterization of NcROP2Fam-1, a member of the ROP2 family of rhoptry proteins in *Neospora caninum* that is targeted by antibodies neutralizing host cell invasion *in vitro*. Parasitology. (2013) 140:1033–50. 10.1017/S0031182013000383 23743240

[23] Debache K Alaeddine F Guionaud C Monney T Müller J Strohbusch M. Vaccination with recombinant NcROP2 combined with recombinant NcMIC1 and NcMIC3 reduces cerebral infection and vertical transmission in mice experimentally infected with *Neospora caninum* tachyzoites. Int J Parasitol. (2009) 39:1373–84. 10.1016/j.ijpara.2009.04.006 19447110

[24] Kato T Otsuki T Yoshimoto M Itagaki K Kohsaka T Matsumoto Y. *Bombyx mori* nucleopolyhedrovirus displaying *Neospora caninum* antigens as a vaccine candidate against *N. caninum* infection in mice. Mol Biotechnol. (2014) 57:145–54. 10.1007/s12033-014-9810-9 25307182

[25] Monney T Grandgirard D Leib SL Hemphill A. Use of a Th1 stimulator adjuvant for vaccination against *Neospora caninum* infection in the pregnant mouse model. Pathogens. (2013) 2:193–208. 10.3390/pathogens2020193 25437035 PMC4235717

[26] Srinivasan S Müller J Suana A Hemphill A. Vaccination with microneme protein NcMIC4 increases mortality in mice inoculated with *Neospora caninum*. J Parasitol. (2007) 93:1046–55. 10.1645/GE-1181R1.1 18163338

[27] Allahyari M Mohabati R Amiri S Rastaghi ARE Babaie J Mahdavi M. Synergistic effect of rSAG1 and rGRA2 antigens formulated in PLGA microspheres in eliciting immune protection against *Toxoplasma gondii*. Exp Parasitol. (2016) 170:236–46. 10.1016/j.exppara.2016.09.008 27663469

[28] Huang P Liao M Zhang H Lee EG Nishikawa Y Xuan X. Dense-granule protein NcGRA7, a new marker for the serodiagnosis of *Neospora caninum* infection in aborting cows. Clin Vaccine Immunol. (2007) 14:1640–3. 10.1128/CVI.00251-07 17959821 PMC2168391

[29] Jin C Yu L Wang Y Hu S Zhang S. Evaluation of *Neospora caninum* truncated dense granule protein 2 for serodiagnosis by enzyme-linked immunosorbent assay in dogs. Exp Parasitol. (2015) 157:88–91. 10.1016/j.exppara.2015.07.003 26172405

[30] Hiszczyńska-Sawicka E Oledzka G Holec-Gasior L Li H Xu JB Sedcole R. Evaluation of immune responses in sheep induced by DNA immunization with genes encoding GRA1, GRA4, GRA6 and GRA7 antigens of *Toxoplasma gondii*. Vet Parasitol. (2011) 177:281–9. 10.1016/j.vetpar.2010.11.047 21251760

[31] Atkinson RA Ryce C Miller CM Balu S Harper PA Ellis JT. Isolation of *Neospora caninum* genes detected during a chronic murine infection. Int J Parasitol. (2001) 31:67–71. 10.1016/s0020-7519(00)00153-3 11165273

[32] Leineweber M Spekker-Bosker K Ince V Schares G Hemphill A Eller SK. First characterization of the *Neospora caninum* dense granule protein GRA9. Biomed Res Int. (2017) 2017:746437. 10.1155/2017/6746437 29259983 PMC5702412

[33] Liu G Cui X Hao P Yang D Liu J Liu Q. GRA 14, a novel dense granule protein from *Neospora caninum*. Acta Biochim Biophys Sin. (2013) 45:607–9. 10.1093/abbs/gmt036 23722878

[34] Yang CS Liu J Ma L Zhang XC Zhang X Zhou BX. NcGRA17 is an important regulator of parasitophorous vacuole morphology and pathogenicity of *Neospora caninum*. Vet Parasitol. (2018) 264:26–34. 10.1016/j.vetpar.2018.03.018 30503087

[35] Wang WR Gong PT Wang P Dong JQ Wang XC Yang. A novel dense granule protein NcGRA23 in *Neospora caninum*. Acta Biochim Biophys Sin. (2018) 50:727–9. 10.1093/abbs/gmy054 29860400

[36] Wang XC Gong PT Zhang X Li S Lu XY Zhao CY. NLRP3 inflammasome participates in host response to *Neospora caninum* infection. Front Immunol. (2018) 9:1791. 10.3389/fimmu.2018.01791 30105037 PMC6077289

[37] Hemphill A Aguado-Martínez A Müller J. Approaches for the vaccination and treatment of *Neospora caninum* infections in mice and ruminant models. Parasitology. (2015) 143:245–59. 10.1017/S0031182015001596 26626124

[38] Barling KS Lunt DK Graham SL Choromanski LJ. Evaluation of an inactivated *Neospora caninum* vaccine in beef feedlot steers. J Am Vet Med Assoc. (2003) 222:624–7. 10.2460/javma.2003.222.624 12619843

[39] Romero JJ Perez E Frankena K. Effect of a killed whole *Neospora caninum* tachyzoite vaccine on the crude abortion rate of Costa Rican dairy cows under field conditions. Vet Parasitol. (2004) 123:149–59. 10.1016/j.vetpar.2004.06.016 15325041

[40] Weston JF Heuer C Williamson NB. Efficacy of a *Neospora caninum* killed tachyzoite vaccine in preventing abortion and vertical transmission in dairy cattle. Prev Vet Med. (2012) 103:136–44. 10.1016/j.prevetmed.2011.08.010 21925752

[41] Reichel MP Moore DP Hemphill A Ortega-Mora LM Dubey JP Ellis JT. A live vaccine against *Neospora caninum* abortions in cattle. Vaccine. (2015) 33:1299–301. 10.1016/j.vaccine.2015.01.064 25659274

[42] Tartarelli I Tinari A Possenti A Cherchi S Falchi M Dubey JP. During host cell traversal and cell-to-cell passage, *Toxoplasma gondii* sporozoites inhabit the parasitophorous vacuole and posteriorly release dense granule protein-associated membranous trails. Int J Parasitol. (2020) 50:1099–115. 10.1016/j.ijpara.2020.06.012 32882286

[43] Mercier C Dubremetz JF Rauscher B Lecordier L Sibley LD Cesbron-Delauw MF. Biogenesis of nanotubular network in Toxoplasma parasitophorous vacuole induced by parasite proteins. Mol Biol Cell. (2002) 13:2397–409. 10.1091/mbc.E02-01-0021 12134078 PMC117322

[44] Bittame A Effantin G Pètre G Ruffiot P Travier L Schoehn G. *Toxoplasma gondii*: biochemical and biophysical characterization of recombinant soluble dense granule proteins GRA2 and GRA6. Biochem Biophy Res Commun. (2015) 459:107–12. 10.1016/j.bbrc.2015.02.078 25712518

[45] Romano JD Nolan SJ Porter C Ehrenman K Hartman EJ Hsia R. The parasite toxoplasma sequesters diverse Rab host vesicles within an intravacuolar network. J Cell Biol. (2017) 216:4235–54. 10.1083/jcb.201701108 29070609 PMC5716271

[46] Nishikawa Y Zhang H Ikehara Y Kojima N Xuan X Yokoyama N. Immunization with oligomannose-coated liposome-entrapped dense granule protein 7 protects dams and offspring from *Neospora caninum* infection in mice. Clin Vaccine Immunol. (2009) 16:792–7. 10.1128/CVI.00032-09 19357313 PMC2691053

[47] Zhang Z Li Y Liang Y Wang S Xie Q Nan X. Molecular characterization and protective immunity of rhoptry protein 35 (ROP35) of *Toxoplasma gondii* as a DNA vaccine. Vet Parasitol. (2018) 260:12–21. 10.1016/j.vetpar.2018.06.016 30197008

[48] Quan JH Chu JQ Ismail HAHA Zhou W Jo EK Cha GH. Induction of protective immune responses by a multiantigenic DNA vaccine encoding GRA7 and ROP1 of *Toxoplasma gondii*. Clin Vaccine Immunol. (2012) 19:666–74. 10.1128/CVI.05385-11 22419676 PMC3346315

[49] Cao A Liu Y Wang J Li X Wang S Zhao Q. *Toxoplasma gondii*: vaccination with a DNA vaccine encoding T- and B-cell epitopes of SAG1, GRA2, GRA7 and ROP16 elicits protection against acute toxoplasmosis in mice. Vaccine. (2015) 33:6757–62. 10.1016/j.vaccine.2015.10.077 26518401

[50] Gong PT Cao LL Guo YB Dong H Yuan SX Yao XH. *Toxoplasma gondii*: protective immunity induced by a DNA vaccine expressing GRA1 and MIC3 against toxoplasmosis in BALB/c mice. Exp Parasitol. (2016) 166:131–6. 10.1016/j.exppara.2016.04.003 27059254

[51] Zhu WN Wang JL Chen K Yue DM Zhang XX Huang SY. Evaluation of protective immunity induced by DNA vaccination with genes encoding *Toxoplasma gondii* GRA17 and GRA23 against acute toxoplasmosis in mice. Exp Parasitol. (2017) 179:20–7. 10.1016/j.exppara.2017.06.002 28625894

[52] Ahmadpour E Sarvi S Soteh MBH Sharif M Rahimi MT Valadan R. Evaluation of the immune response in BALB/c mice induced by a novel DNA vaccine expressing GRA14 against *Toxoplasma gondii*. Parasite Immunol. (2017) 39:e12419. 10.1111/pim.12419 28186325

[53] Khan IA Schwartzman JD Fonseka S Kasper LH. *Neospora caninum*: role for immune cytokines in host immunity. Exp Parasitol. (1997) 85:24–34. 10.1006/expr.1996.4110 9024199

[54] Nishikawa Y Tragoolpua K Inoue N Makala L Nagasawa H Otsuka H. In the absence of endogenous gamma interferon, mice acutely infected with *Neospora caninum* succumb to a lethal immune response characterized by inactivation of peritoneal macrophages. Clin Diagn Lab Immunol. (2001) 8:811–6. 10.1128/CDLI.8.4.811-817.2001 11427432 PMC96148

[55] Haldar AK Saka HA Piro AS Dunn JD Henry SC Taylor GA. IRG and GBP host resistance factors target aberrant, “non-self” vacuoles characterized by the missing of “self” IRGM proteins. PLoS Pathog. (2013) 9:e1003414. 10.1371/journal.ppat.1003414 23785284 PMC3681737

[56] Nishikawa Y. Towards a preventive strategy for neosporosis: challenges and future perspectives for vaccine development against infection with *Neospora caninum*. J Vet Med Sci. (2017) 79:1374–80. 10.1292/jvms.17-0285 28690279 PMC5573824

