# Supplementary figures and images for: Immune Protective Evaluation Elicited by DNA Vaccination With Neospora caninum Dense Granules Proteins in Mice
Source: Front Vet Sci. 2021 Feb 26;8:638067. doi: 10.3389/fvets.2021.638067 (PMC7953147; doi:10.3389/fvets.2021.638067)

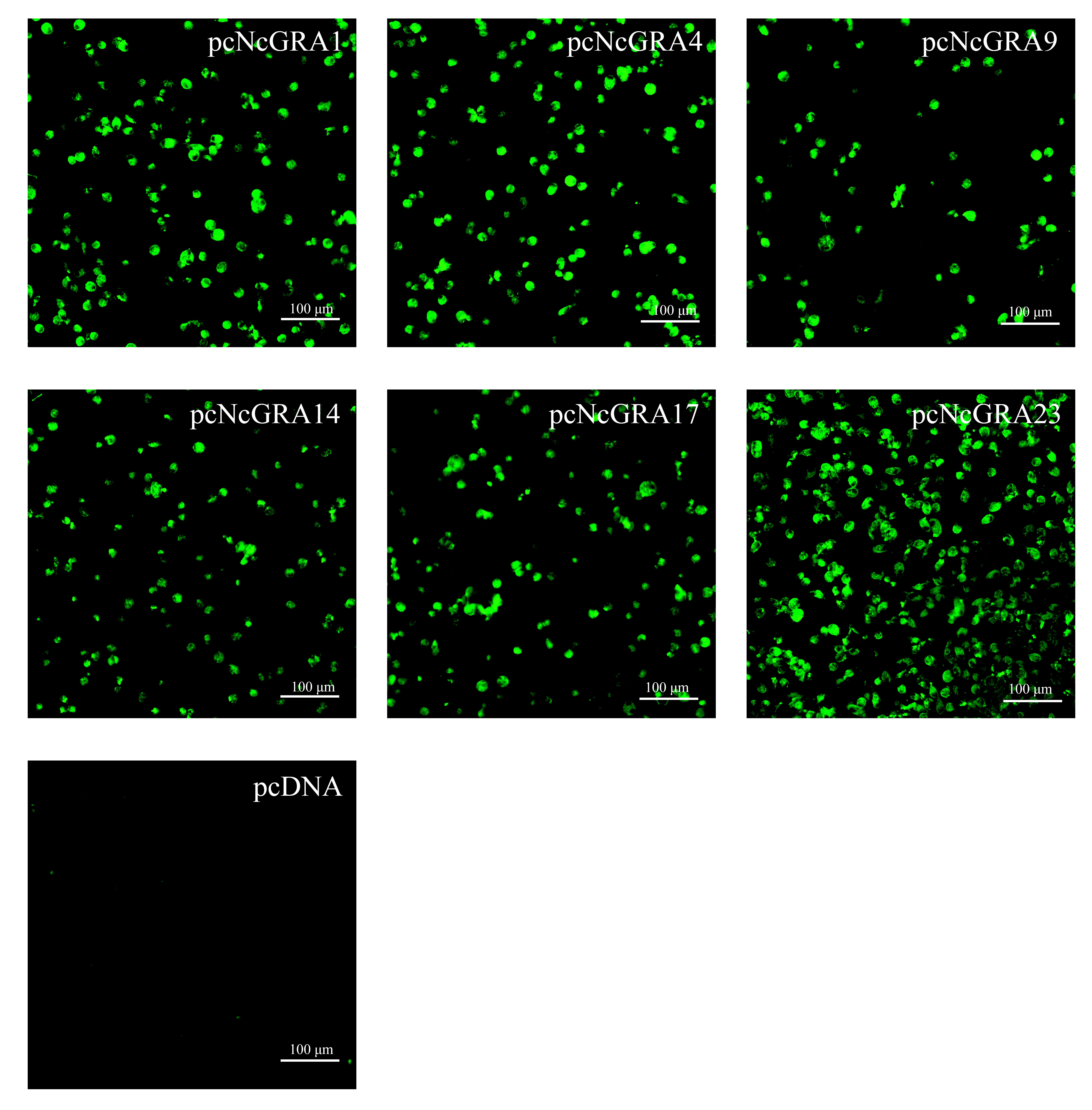

Supplement: Supplementary file 1 [file Image_1.TIF]
